# Suprathreshold Heat Pain Response Predicts Activity-Related Pain, but Not Rest-Related Pain, in an Exercise-Induced Injury Model

**DOI:** 10.1371/journal.pone.0108699

**Published:** 2014-09-29

**Authors:** Rogelio A. Coronado, Corey B. Simon, Carolina Valencia, Jeffrey J. Parr, Paul A. Borsa, Steven Z. George

**Affiliations:** 1 Department of Physical Therapy, University of Florida, Gainesville, Florida, United States of America; 2 Department of Applied Medicine and Rehabilitation, Indiana State University, Terra Haute, Indiana, Unites States of America; 3 Department of Sports Medicine and Nutrition, University of Pittsburgh, Pittsburgh, Pennsylvania, United States of America; 4 Department of Applied Physiology and Kinesiology, University of Florida, Gainesville, Florida, United States of America; 5 Center for Pain Research and Behavioral Health, University of Florida, Gainesville, Florida, United States of America; University of Washington, United States of America

## Abstract

Exercise-induced injury models are advantageous for studying pain since the onset of pain is controlled and both pre-injury and post-injury factors can be utilized as explanatory variables or predictors. In these studies, rest-related pain is often considered the primary dependent variable or outcome, as opposed to a measure of activity-related pain. Additionally, few studies include pain sensitivity measures as predictors. In this study, we examined the influence of pre-injury and post-injury factors, including pain sensitivity, for induced rest and activity-related pain following exercise induced muscle injury. The overall goal of this investigation was to determine if there were convergent or divergent predictors of rest and activity-related pain. One hundred forty-three participants provided demographic, psychological, and pain sensitivity information and underwent a standard fatigue trial of resistance exercise to induce injury of the dominant shoulder. Pain at rest and during active and resisted shoulder motion were measured at 48- and 96-hours post-injury. Separate hierarchical models were generated for assessing the influence of pre-injury and post-injury factors on 48- and 96-hour rest-related and activity-related pain. Overall, we did not find a universal predictor of pain across all models. However, pre-injury and post-injury suprathreshold heat pain response (SHPR), a pain sensitivity measure, was a consistent predictor of activity-related pain, even after controlling for known psychological factors. These results suggest there is differential prediction of pain. A measure of pain sensitivity such as SHPR appears more influential for activity-related pain, but not rest-related pain, and may reflect different underlying processes involved during pain appraisal.

## Introduction

Pain ratings are commonly queried during the assessment of individuals with musculoskeletal pain. In clinical studies, current or rest-related pain intensity is most often assessed [1], while pain ratings with specific activity (e.g., activity-related pain) are less frequently reported. A recent systematic review of post-surgical trials by Srikandarajah and Gilron [2] found that only 39% (281/726) of published trials included a measurement of activity-related pain as a clinical outcome. Failure to include a measure of activity-related pain is noteworthy as activity-related pain may have a stronger association with functional limitations, or greater sensitivity in assessing therapeutic response, than measures of rest-related pain [3], [4].

Differentiating rest and activity-related pain can be difficult in clinical studies. Exercise-induced injury models may better control comparisons of rest and activity-related pain. In a study by Dannecker and Sluka [5], the authors showed that following eccentric exercise for the elbow flexors, higher pain ratings occurred with elbow extension than at rest. This study is relevant as it suggests exercise-induced injury models can induce higher levels of pain dependent on activity, and may be appropriate models for studying the prediction of rest and activity-related pain.

Predictive models have been generated to determine which factors influence pain outcomes following exercise-induced injury [6]–[9]. Similar to clinical studies, most exercise-induced injury studies focus on predicting rest-related pain rather than activity-related pain. Factors shown to be predictive of rest-related pain are predominantly psychological in nature, such as pain catastrophizing [9], [10]. However, current limitations in the prediction of pain following exercise-induced injury are twofold. First, it is unclear whether psychological factors are predictors of activity-related pain. Second, measures of pain sensitivity have not been widely considered within predictive models for pain following exercise-induced injury [6], [7]. Pain sensitivity measures are thought to reflect pain processing within the nervous system and are receiving attention as potential prognostic variables [11], [12]. Pain sensitivity can potentially improve prediction models that already include psychological factors and, therefore, may allow better evaluation of different processes relevant to the perception of rest or activity- related pain. Furthermore, pain sensitivity, if found to be an important predictor, could be considered a relevant factor to include in clinical assessment, especially as it is not commonly assessed in a clinical context [13].

Thus, further investigation is needed to ascertain the influence pain sensitivity has on an individual's pain perception at rest or during activity. Specifically, we sought primarily to assess the predictive ability of 1) baseline, pre-injury pain sensitivity on post-injury rest and activity-related pain at 48 and 96-hours and 2) 48-hours post-injury pain sensitivity on post-injury rest and activity-related pain at 96-hours. We used an exercise-induced injury model in a group of healthy volunteers, as these models are standardized induced-pain methods to study the development and progression of chronic pain. In these models, the overall pain experience is shorter in duration, yet generates similar pain and impairment characteristics observed in clinical pain conditions [14]–[16]. Moreover, the influence of both pre-injury (e.g., pre-clinical) and post-injury (e.g., clinical) factors on the painful episode can be examined as the onset of injury is controlled within the model. We hypothesized that pain sensitivity factors would be predictive of rest and activity-related pain, even after controlling for psychological factors. We did not have a specific hypothesis on a differential influence of pain sensitivity on pain outcome.

## Materials and Methods

### Ethics Statement

This study was approved by the University of Florida Institutional Review Board. Prior to enrollment, all participants provided written informed consent.

### Participants

Participants were recruited from the campus of the University of Florida and the surrounding community. All participants were between the ages of 18 and 85 years old and not performing strength training exercises for the upper extremity either currently or during the previous 6 weeks. Participants were not included if they 1) were currently experiencing neck or shoulder pain, 2) had neurological impairments of the upper extremity, such as loss of sensation, muscle weakness, or reflex changes, 3) were currently taking pain medication, or 4) had previous shoulder surgery.

### Procedure

After providing written informed consent at the initial assessment session, all participants completed standardized questionnaires and underwent pain sensitivity testing. Testing was conducted in the same private laboratory environment controlled for room temperature and humidity, and where distractions were minimized. Next, participants performed an eccentric exercise protocol on their dominant shoulder to induce pain [8] using a Kin-Com isokinetic dynamometer (Chattanooga Group, Chattanooga, TN). Participants were seated according to manufacturer's recommendations and straps were affixed across the torso to minimize trunk motion. The dominant shoulder was placed in a standardized position within the scapular plane. After determining maximum voluntary isometric contraction (MVIC) for each individual, participants completed repetitions of isokinetic concentric/eccentric external rotation actions to induce shoulder pain. Specifically, participants performed a standard bout of 3 sets of 10 repetitions at 60°/sec with a goal of fatiguing participants so they generated 50% or less of their initial MVIC. If necessary, additional repetitions (e.g., 1 set of 8 repetitions) were performed until the standard fatigue level was reached. After completing the initial session which lasted approximately one hour, participants were then scheduled for 2 follow-up sessions. Follow-up sessions occurred at 48- and 96-hours after the initial session, where repeat measurements of psychological variables, pain sensitivity, and outcome occurred at 48-hours and outcome alone at 96-hours.

### Measures

#### Demographics

Demographic information included age, sex, height, weight, and dominant arm. Height and weight information were used to compute body mass index (BMI) as a covariate for exercise-induced injury.

#### Shoulder Pain Intensity

Using a 101-point numeric pain rating scale (0 = “no pain,” 100 = “worst pain imaginable”), participants verbally indicated their intensity of shoulder pain [17]. Shoulder pain intensity was asked during three states: 1) at rest (e.g., upper extremity held at side); 2) during active motion (e.g., shoulder abduction); 3) during isometric activation (e.g., resisted external rotation).

#### Pain Catastrophizing

Pain catastrophizing was assessed using the Pain Catastrophizing Scale (PCS) as this variable has been shown to influence pain-related outcomes [9],[18],[19]. The PCS is a 13-item questionnaire (score ranges from 0 to 52) that measures thoughts on various pain experiences where higher scores reflect higher levels of pain catastrophizing [20].

#### Fear of Movement

Fear of movement was assessed using the shortened version of the Tampa Scale of Kinesiophobia (TSK-11) and has shown to be associated with shoulder injury outcome [21], [22]. The TSK-11 is a 17-item questionnaire (score ranges from 11 to 44) that measures fear of movement where higher scores reflect higher levels of fear of movement [23].

#### Pressure Pain Threshold

Pressure pain threshold (PPT) was measured at the tip of the acromion of the dominant shoulder using a hand-held algometer (Pain Diagnostics & Treatment, Great Neck, NY) with a 1 cm^2^ diameter probe. PPT is a single stimulus threshold measure of static pain sensitivity, and is commonly used in laboratory and clinical research trials [24]. Our assessment of PPT for the shoulder undergoing exercise-induced injury is a marker of local pain sensitivity. [25], [26] A trained examiner applied a standard pressure force with a target rate of approximately 1 kg/sec until the pressure sensation was first reported by the participant to be painful [25]–[28]. Lower PPT values were indicative of higher pain sensitivity. A total of 3 trials were obtained with rest in between each testing trial. The average of the 3 trials was used for analyses.

#### Suprathreshold Heat Pain Response

Suprathreshold heat pain response (SHPR) was measured at the thenar aspect of both hands using a contact thermode with 2.5 cm^2^ surface area connected to a computer-controlled PATHWAY Model Contact Heat Evoked Potential Stimulator (CHEPS) (Medoc Advanced Medical Systems). SHPR is a dynamic measure of pain sensitivity and thought to be predominantly mediated by C-fiber activity [24], [29]. A series of 5 consecutive heat pulses at a rate of 30°C/sec with interstimulus interval of 2.5 seconds was delivered with a peak temperature of 48°C. We used 48°C as this temperature elicits a moderate level of pain (e.g., pain ratings near 50/100 on pain rating scale) for a majority of participants [30]. Each heat pulse was rated by the participant using the same 101-point numeric pain rating scale. SHPR was identified as the pain intensity rating of the fifth heat pulse in a train of 5 heat pulses and has been used as a clinically meaningful dynamic measure of pain sensitivity [30], [31]. Higher pain ratings on SHPR indicate higher pain sensitivity. The average SHPR rating of both hands was used for analyses.

#### Conditioned Pain Modulation

Conditioned pain modulation (CPM) was measured using the same CHEPS setup as SHPR. The CPM protocol included a measured response of a test stimulus before and after application of a conditioning stimulus [32]. The test stimulus was delivered to the thenar aspect of the non-dominant hand and involved a sequence of 5 heat pulses at a temperature that induced a moderate level of pain intensity (e.g., 50/100 on pain scale) with the same parameters as SHPR. Participants rated each heat pulse with the same 101-point numeric pain rating scale used in the SHPR protocol. The pain rating of the fifth heat pulse was used as the test stimulus response. For the conditioning stimulus, participants immersed their dominant hand into a cold water bath for up to 60 seconds. The water temperature was maintained at a constant temperature of 8°C using a refrigeration unit (NESLAB RTE 7 Digital One, Thermo Scientific Co., Massachusetts, USA) which circulated water to prevent warming. Participants were instructed to keep their hand in the cold water bath for a minimum of 30 seconds, at which time they could remove their hand, if needed. After the 60 second cold water time period, a second bout of the same test stimulus was conducted. CPM was computed as the absolute difference in SHPR before and after the conditioning stimulus [32]. Like SHPR, CPM is a dynamic measure of pain sensitivity and indicative of central sensitivity. Moreover, CPM is a behavioral correlate of endogenous pain inhibition [33]. Larger differences (e.g., larger reductions in SHPR) were indicative of greater degrees of pain modulation.

### Data Analysis

We used IBM SPSS Statistics for Windows (version 21.0, SPSS, Inc., Chicago, IL) for all data analyses. Alpha was set a priori at the 0.05 level for statistical significance.

Descriptive statistics were computed for all pertinent baseline and follow-up measures. To assess whether the exercise-induced injury model induced changes in relevant factors, baseline and 48-hour psychological variables (PCS, TSK-11) and pain sensitivity (PPT, SHPR, CPM) comparisons were made using a paired samples t-test.

The primary dependent variables (e.g., outcomes) for all analyses were the exercise-induced 1) rest-related shoulder pain (pain intensity with shoulder at rest); 2) shoulder pain with motion (pain intensity during active shoulder abduction); and 3) isometric shoulder pain (pain during isometric resisted external rotation of the shoulder). The latter two dependent variables were considered measures of activity-related pain. These variables were measured at 48- and 96-hours after exercise-induced shoulder pain.

Zero-order correlation coefficients were computed for independent variables (e.g., predictors) measured at baseline (pre-injury: age, sex, BMI, PCS, TSK-11, PPT, SHPR, CPM) with dependent variables at 48- and 96-hours, and independent variables measured at 48-hours (post-injury: PCS, TSK-11, PPT, SHPR, CPM) with dependent variables at 96-hours. All variables were analyzed as continuous variables, except for sex which was a dichotomous variable (0 for female, 1 for male). These correlations coefficients express the simple relationship between each independent and dependent variable.

Subsequently, we examined the unique relationship between independent and dependent variables after controlling for other variables, using hierarchical linear regression. Hierarchical linear regression modeling allows us to account for other factors in an a-priori, theoretical fashion. Separate hierarchical linear regression models were created using 1) baseline, pre-injury independent variables for each of the three dependent variables at 48- and 96-hours and 2) baseline demographic (age, sex, BMI) and 48-hour, post-injury independent variables (PCS, TSK-11, PPT, SHPR, CPM) for each of the three dependent variables at 96-hours.

In each of the hierarchical linear regression models, demographic variables of age, sex, and BMI were entered into the first block, psychological variables of PCS and TSK-11 were entered into the second block, and pain sensitivity variables of PPT, SHPR, and CPM were entered into the third and final block. In the 96-hour prediction model, each respective independent variable outcome measured at 48-hours was entered in the first block. We built these models in this fashion to assess the unique contribution of pain sensitivity factors after accounting for relevant demographic and psychological variables.

Correlation values between independent variables were examined prior to entering into regression models in order to prevent significant inter-correlation (i.e., multicollinearity). An a-priori correlation of 0.7 or greater was used to reflect potential multicollinearity [34]. Additional multicollinearity tests were tolerance and variance inflation, which should be greater than.2 and less than 10, respectively [35]. All tests indicated a lack of multicollinearity, and therefore, confirmed the stability of our models. Overall model statistics were examined along with changes in r-squared and p-value for each model step. Relative individual predictor strength was assessed with β estimates.

### Sample Size Determination

To determine the minimum acceptable sample size needed for these analyses, we used estimation criteria from Green [36], as well as the general rule of thumb of 10–15 cases per predictor, as guidelines [35], [37]. Since we were interested in assessing both the overall fit of our regression modeling and testing individual predictors (n = 8) we used the larger of the two sample sizes needed from Green's criteria. In this case, the minimum total sample size needed was 114 participants and was approximate to the more stringent rule of thumb criteria of 120 participants (e.g., 15 cases per predictor with 8 predictors).

## Results

### Sample Characteristics

Descriptive data from 143 individuals who participated in this study are presented in Table 1. All enrolled participants, except for one (illness), completed study. Participants ranged in age from 18 to 58 years (mean age = 23.7 years, SD = 6.7 years) and the 59% were female (85 female, 58 male). Comparison of baseline and 48-hour values for psychological variables and pain sensitivity showed that exercise-induced shoulder pain resulted in significantly lower PPT (higher local pain sensitivity) and PCS (lower pain catastrophizing) values and higher TSK-11 values (higher fear of movement) (p<0.05).

**Table 1:** Baseline, 48-hour, and 96-hour Descriptive Data (N = 143).

	Pre-injury	Post-injury	
Variable	Baseline	48-hour	96-hour
**Demographic**			
Age (years)	23.7 (6.7)	-	-
Sex (N of females)	85	-	-
Dominant arm (N of right)	126	-	-
BMI	23.5 (4.0)		
**Psychological**			
PCS	9.9 (7.9)	8.0 (8.0)	-
TSK-11	18.0 (4.4)	18.8 (5.0)	-
**Pain Sensitivity**			
PPT (kg)	5.5 (2.0)	4.8 (1.8)	-
SHPR (x/100)	23.2 (24.4)	20.9 (22.7)	-
CPM (x/100)	−8.6 (12.8)	−7.8 (13.6)	-
**Shoulder Pain Intensity (x/100)**			
At Rest	-	22.2 (20.5)	7.5 (10.1)
With Active Motion	-	15.5 (16.4)	4.2 (7.4)
With Isometric Contraction	-	20.6 (18.8)	10.5 (16.3)

Values are expressed as mean (SD) unless otherwise indicated.

Abbreviations: BMI = body mass index, CPM = conditioned pain modulation, N = number, PCS = Pain Catastrophizing Scale, PPT = pressure pain threshold, SHPR = suprathreshold heat pain response, TSK-11 = Tampa Scale of Kinesiophobia.

### Association between Predictor and Outcome Variables

Table 2 lists correlation coefficients for each pre-injury and post-injury variable and 48- and 96-hour pain outcome. For pain sensitivity predictors, pre-injury SHPR was positively associated with both measures of activity-related pain at 48- and 96-hours (p<0.05), but not rest-related pain at these time points (p>0.05). Furthermore, post-injury SHPR, along with post-injury PPT, was associated with 96-hour activity-related pain (p<0.05). The relationship of SHPR and PPT with pain outcome was such that the more pain sensitive an individual was (e.g., higher SHPR or lower PPT), the higher the report of shoulder pain.

**Table 2:** Correlation of Pre-Injury and Post-Injury Predictors with Shoulder Pain Outcome at 48 and 96-Hours.

	48-Hours			96-Hours		
		Activity-Related Pain			Activity-Related Pain	
Variable	Rest-Related Pain	Motion Pain	Isometric Pain	Rest-Related Pain	Motion Pain	Isometric Pain
**Pre-injury**						
Age	−0.014	0.011	−0.053	−0.123	−0.084	−0.096
Sex	−0.171*	0.066	−0.039	−0.066	0.050	0.040
BMI	−0.051	0.033	−0.062	−0.020	0.042	0.005
PCS	0.096	0.125	0.128	0.175*	0.179*	0.145
TSK-11	−0.015	−0.044	−0.003	0.073	0.073	0.091
PPT	−0.131	−0.076	−0.162*	−0.048	−0.051	−0.079
SHPR	0.045	0.251*	0.437*	0.073	0.222*	0.320*
CPM	0.091	−0.051	−0.118	−0.056	−0.197*	−0.039
**Post-Injury**						
48 Pain	-	-	-	0.423*	0.568*	0.670*
PCS	-	-	-	0.253*	0.176*	0.095
TSK-11	-	-	-	0.139	0.201*	0.050
PPT	-	-	-	−0.129	−0.233*	−0.203*
SHPR	-	-	-	0.162*	0.286*	0.418*
CPM	-	-	-	−0.023	−0.087	0.017

*p-value<0.05.

Values are correlation coefficients.

Abbreviations: BMI = body mass index, CPM = conditioned pain modulation, PCS = Pain Catastrophizing Scale, PPT = pressure pain threshold, SHPR = suprathreshold heat pain response, TSK = Tampa Scale of Kinesiophobia.

### Prediction of Rest- and Activity-Related Pain at 48 Hours

Table 3 includes results from the hierarchical regression models predicting 48-hour rest-related shoulder pain and shoulder pain with activity using pre-injury predictors. The final regression model for 48-hour rest-related shoulder pain indicated that none of the covariate variables were predictive of outcome (F_8,133_ = 1.145, p = 0.338, r^2^ = 0.064). In contrast, the final regression models for shoulder pain with motion (F_8,132_ = 2.360, p<0.05) and isometric shoulder pain (F_8,130_ = 5.159, p<0.001) demonstrated predictive ability explaining 12.5% and 24.1% of the variance in pain outcome, respectively. In these models, pre-injury SHPR (β = 0.285, p<0.05) was the strongest predictor of 48-hour shoulder pain with motion, even after accounting for psychology (PCS: β = 0.209, p<0.05). In addition, pre-injury SHPR (β = 0.472, P<0.05) was the sole predictor of 48-hour isometric shoulder pain.

**Table 3:** Hierarchical Regression Models Predicting 48-hour Shoulder Pain at Rest, with Motion, and with Isometric Action Using Baseline, Pre-injury Predictors.

					Activity-Related Pain							
	Rest-Related Pain				Motion Pain				Isometric Pain			
	Beta	P	Δ R^2^	P	Beta	P	Δ R^2^	P	Beta	P	Δ R^2^	P
**1**			0.027	0.281			0.006	0.850			0.005	0.889
Age	0.025	0.785			<0.001	0.996			−0.027	0.771		
Sex	−0.163	0.065			0.071	0.423			−0.011	0.902		
BMI	−0.016	0.867			0.013	0.890			−0.049	0.605		
**2**			0.020	0.249			0.043	0.232			0.029	0.461
Age	0.061	0.512			0.054	0.564			0.013	0.893		
Sex	−0.157	0.076			0.079	0.367			−0.005	0.955		
BMI	−0.021	0.819			0.004	0.968			−0.053	0.577		
PCS	0.170	0.095			**0.247**	**0.015**			**0.202**	**0.048**		
TSK-11	−0.099	0.323			−0.149	0.135			−0.088	0.382		
**3**			0.017	0.338			0.076	0.021			0.207	<0.001
Age	0.071	0.449			0.054	0.552			0.011	0.899		
Sex	−0.137	0.140			0.107	0.232			0.040	0.632		
BMI	0.017	0.855			0.051	0.579			0.023	0.789		
PCS	0.170	0.100			**0.209**	**0.036**			0.133	0.149		
TSK-11	−0.074	0.466			−0.144	0.142			−0.080	0.378		
PPT	−0.057	0.533			−0.061	0.494			−0.084	0.311		
SHPR	0.083	0.384			**0.285**	**0.002**			**0.472**	**<0.001**		
CPM	0.128	0.186			0.041	0.665			0.056	0.527		

All predictor variables measured at baseline (pre-injury).

Abbreviations: BMI = body mass index, CPM = conditioned pain modulation, PCS = Pain Catastrophizing Scale, P = p-value, PPT = pressure pain threshold, SHPR = suprathreshold heat pain response, TSK = Tampa Scale of Kinesiophobia.

### Prediction of Rest- and Activity-Related Pain at 96 Hours

Table 4 includes results from the hierarchical regression models predicting 96-hour resting shoulder pain and shoulder pain with activity using pre-injury predictors. The final regression model predicting 96-hour rest-related shoulder pain was not statistically significant, and therefore, was not predictive of outcome (F_8,131_ = 0.683, p = 0.706, r^2^ = 0.040). The final regression models for shoulder pain with motion (F_8,130_ = 2.259, p = 0.027) and isometric shoulder pain (F_8,128_ = 3.102, p = 0.003) demonstrated predictive ability, explaining 12.2% and 16.2% of the variance in pain outcome, respectively. Despite lack of significance, pre-injury SHPR (β = 0.172, p = 0.067) was the strongest predictor of 96-hour shoulder pain with motion. Moreover, pre-injury SHPR was a unique predictor (β = 0.387, p<0.001) of 96-hour isometric shoulder pain.

**Table 4:** Hierarchical Regression Models Predicting 96-hour Shoulder Pain at Rest, with Motion, and with Isometric Action Using Baseline, Pre-Injury Predictors.

					Activity-Related Pain							
	Rest-Related Pain				Motion Pain				Isometric Pain			
	Beta	P	Δ R^2^	P	Beta	P	Δ R^2^	P	Beta	P	Δ R^2^	P
**1**			0.022	0.379			0.016	0.541			0.014	0.607
Age	−0.138	0.134			−0.119	0.201			−0.116	0.215		
Sex	−0.068	0.446			0.060	0.506			0.061	0.501		
BMI	0.050	0.592			0.070	0.463			0.031	0.746		
**2**			0.016	0.379			0.036	0.205			0.025	0.393
Age	−0.111	0.240			−0.071	0.455			−0.084	0.382		
Sex	−0.058	0.512			0.070	0.430			0.068	0.452		
BMI	0.052	0.580			0.072	0.445			0.037	0.699		
PCS	0.139	0.179			**0.204**	**0.048**			0.139	0.179		
TSK-11	−0.018	0.859			−0.012	0.900			0.038	0.702		
**3**			0.002	0.706			0.070	0.027			0.124	0.003
Age	−0.111	0.249			−0.087	0.348			−0.092	0.312		
Sex	−0.049	0.601			0.094	0.303			0.102	0.257		
BMI	0.059	0.541			0.121	0.193			0.088	0.340		
PCS	0.134	0.203			0.166	0.100			0.100	0.311		
TSK-11	−0.016	0.876			−0.039	0.694			0.054	0.573		
PPT	−0.024	0.794			−0.046	0.604			−0.040	0.652		
SHPR	0.033	0.736			0.172	0.067			**0.387**	**<0.001**		
CPM	0.005	0.960			−0.145	0.123			0.127	0.172		

All predictor variables measured at baseline (pre-injury).

Abbreviations: BMI = body mass index, CPM = conditioned pain modulation, PCS = Pain Catastrophizing Scale, P = p-value, PPT = pressure pain threshold, SHPR = suprathreshold heat pain response, TSK = Tampa Scale of Kinesiophobia.

Table 5 includes results from the hierarchical regression models predicting 96-hour rest and activity-related pain using 48-hour post-injury predictors. The final regression model for 96-hour rest-related shoulder pain was predictive of outcome (F_9,129_ = 4.700, p<0.001), explaining 24.7% of the variance. The final regression models for shoulder pain with motion (F_9,128_ = 9.338, p<0.001) and isometric shoulder pain (F_9,126_ = 13.266, p<0.001) were also predictive, explaining 39.6% and 48.7% of the variance in pain outcome, respectively. Pain outcome measured at 48-hours was the strongest predictor across all models (i.e., rest pain at 48-hours for rest pain at 96-hours, etc.), however, post-injury PPT (β = −0.164, p<0.05) and post-injury SHPR (β = 0.154, p<0.05) also predicted 96-hour shoulder pain with motion. Furthermore, post-injury SHPR (β = 0.164, p<0.05) was a predictor of 96-hour isometric shoulder pain.

**Table 5:** Hierarchical Regression Models Predicting 96-hour Shoulder Pain at Rest, with Motion, and with Isometric Action using 48-Hour, Post-Injury Predictors.

					Activity-Related Pain							
	Rest-Related Pain				Motion Pain				Isometric Pain			
	Beta	P	Δ R^2^	P	Beta	P	Δ R^2^	P	Beta	P	Δ R^2^	P
**1**			0.216	<0.001			0.334	<0.001			0.458	<0.001
Age	−0.149	0.074			−0.111	0.150			−0.075	0.284		
Sex	0.001	0.986			0.040	0.596			0.071	0.295		
BMI	0.055	0.520			0.067	0.395			0.063	0.379		
48 Pain	**0.445**	**<0.001**			**0.563**	**<0.001**			**0.669**	**<0.001**		
**2**			0.018	<0.001			0.011	<0.001			0.001	<0.001
Age	−0.129	0.123			−0.103	0.190			−0.074	0.300		
Sex	0.009	0.913			0.036	0.629			0.073	0.287		
BMI	0.043	0.614			0.075	0.341			0.059	0.412		
48 Pain	**0.410**	**<0.001**			**0.566**	**<0.001**			**0.667**	**<0.001**		
PCS	0.148	0.122			−0.062	0.482			0.032	0.684		
TSK-11	−0.016	0.866			0.125	0.148			−0.034	0.665		
**3**			0.013	<0.001			0.052	<0.001			0.027	<0.001
Age	−0.129	0.130			−0.106	0.172			−0.086	0.232		
Sex	0.022	0.791			0.100	0.188			0.084	0.234		
BMI	0.051	0.553			0.113	0.150			0.060	0.404		
48 Pain	**0.398**	**<0.001**			**0.497**	**<0.001**			**0.594**	**<0.001**		
PCS	0.146	0.130			−0.070	0.421			0.037	0.638		
TSK-11	−0.010	0.913			0.135	0.110			−0.031	0.689		
PPT	−0.039	0.642			**−0.164**	**0.032**			−0.011	0.874		
SHPR	0.101	0.213			**0.154**	**0.041**			**0.164**	**0.035**		
CPM	0.054	0.499			<0.001	0.996			0.114	0.087		

All predictor variables except age, sex, and BMI measured at 48 hours (post-injury).

Abbreviations: 48 Pain = Pain report of each respective outcome variable at 48 hours, BMI = body mass index, CPM = conditioned pain modulation, PCS = Pain Catastrophizing Scale, P = p-value, PPT = pressure pain threshold, SHPR = suprathreshold heat pain response, TSK = Tampa Scale of Kinesiophobia.

## Discussion

We induced shoulder pain using an eccentric exercise protocol and evaluated the influence of pre-injury and post-injury factors, namely pain sensitivity, on rest and activity-related shoulder pain measured at 48- and 96-hours. We did not observe consistent predictors across both rest and activity-related pain. However, SHPR, a pain sensitivity measure, was a consistent and oft-times the sole predictor of activity-related pain. These findings suggest that rest and activity-related pain may be modulated by different underlying processes and mechanisms [10]. Specifically, the results of our study indicate that activity-related pain is more likely to be modulated by factors related to pain sensitivity. The relevance of these findings for pain research and clinical practice is further considered in subsequent sections.

### Relevance to Pain Research

Exercise-induced injury paradigms like the one in our study have been used to model the pain experience [5]–[9], [11], [15], [16], [18], [19], [38]–[42]. In general, pre-injury psychological factors have been shown to be predictive of post-injury resting pain reports [7]–[9], [18], [19]. Pain sensitivity, however, predicted activity-related pain, even after controlling for selected psychological factors. These findings are consistent with results reported by Rakel et al. [10] that examined pre-operative psychological and pain sensitivity predictors of rest and activity-related pain following total knee replacement. These authors found pre-operative pain sensitivity measures (e.g., cutaneous mechanical and thermal threshold) to be predictive of activity-related movement pain, but not rest-related pain. Additionally, PPT had limited predictive ability on either rest or activity-related pain [10], which is concordant with our PPT findings.

Our study advances current evidence by including both static (PPT) and dynamic (SHPR, CPM) pain sensitivity measures in predicting pain. In doing so, we show that a dynamic measure of pain sensitivity, SHPR, is an important predictor of clinical pain outcome in a novel pain model (e.g., exercise-induced shoulder injury model) and corroborates a previously published trial of exercise-induced pain [6], [7]. Bishop et al [6] examined the influence of static and dynamic thermal pain sensitivity on exercise-induced low back pain and reported a significant association between dynamic thermal pain sensitivity (e.g., temporal summation of pain) and daily muscle pain intensity. While the authors did not specifically examine activity-related pain, measures of daily muscle pain intensity may include appraisals of activity-related pain within their metric, especially if activity-related pain coincides with moments of worst pain during the day. This may explain why we found no association between pain sensitivity and resting pain intensity, because resting pain only assesses current pain when no movement is occurring at the shoulder.

The lack of consistent predictors across rest and activity-related pain aligns with current models on the pain experience or pain perception [43], [44]. Pain sensation appraisal is context-dependent and involves influence from cognitive, sensory and affective components, and is likely processed in serial, parallel, and cyclical fashion. Conceptually, individuals may appraise pain differently when at rest and during activity [45], [46], and possible explanations include the following mechanisms. First, rating pain during a controlled activity (i.e., lifting arm in controlled fashion like in our study), may not be as threatening as pain during either unpredictable or uncontrollable moments (e.g., daily events or at rest when not anticipating pain). Second, evoked pain may be seen as a challenge to the sensory system and result in activity-dependent responses. Thus, pain sensitivity may be suited to reflect these underlying activity-dependent states more so than during basal states such as rest.

### Relevance to Clinical Practice

While our findings are consistent with findings across pre-clinical (i.e., healthy participant) and postoperative studies [6], [7], [10], [47]–[49], we used an exercise-induced injury model which is advantageous for the assessment of pre-injury influence. We are unaware of studies that assessed the association between pain sensitivity and the transition of pre-injury to post-injury states and this was one of our primary aims [47], [48]. Measuring pre-injury states is difficult to conduct in clinical studies where the future development of pain is uncertain. While our findings are preliminary, our results suggest that early indication of individuals at risk for persistent activity-related pain may be identified by pre-injury pain sensitivity. This has potential implications for clinical management or screening as pain sensitivity assessment may be an additional tool to predict individuals at risk for chronic pain [47]. Recommendations such as these have been made in populations including postoperative pain [50], [51] and there are ongoing efforts for the identification of pain sensitivity profiles or phenotypes for improved pain management [52]. If our findings are replicated in patients with musculoskeletal pain, then current recommendations may be extended to suggest phenotypes of pain-free individuals at risk of activity-evoked pain after musculoskeletal injury.

In a well-conducted meta-analysis, Hubscher et al [53] reported a weak association between pain sensitivity responses and clinical pain intensity. Rather than reflect poor clinical validity of pain sensitivity, the apparent weak association with pain sensitivity and clinical pain potentially may suggest a more complex relationship between measures of pain sensitivity and clinical outcome. Pain sensitivity, in general, may in fact be weakly associated to resting pain ratings, but may show stronger associations when evoked pain (as occurs during movement) is considered the clinical outcome of interest. Not apparent in the review by Hubscher et al [53] is whether a distinction between rest and activity-related pain was made when assessing outcome. But, perhaps in future clinical studies the utility of pain sensitivity will not be in predicting rest-related pain, but in activity-related pain.

Furthermore, select pain sensitivity responses may yield better association or prediction than others. Hubscher et al [53] reported relatively stronger correlations between clinical pain intensity and dynamic pain sensitivity (e.g., temporal summation), as compared to static pain sensitivity. This is consistent with our current findings where SHPR showed stronger relative prediction than PPT. Interestingly, we did not find CPM to be predictive of activity-related pain and that may be because our exercise-induced injury model is a model of acute pain. Evidence suggests that a lack of inhibitory mechanisms, as inferred through CPM, is more often shown in chronic pain syndromes [54], [55], so these results may be expected and future studies in clinical populations should continue to use CPM until this issue is explored further.

### Study Limitations

This study has several limitations. First, we standardized the initial bout of exercise for injury induction, but allowed continued exercise performance if our fatigue criterion was not met. Thus, we did not include the dosage of exercise to induce injury into our models for those individuals requiring a larger volume to induce the same injury criterion. Second, our study results can only be generalized to a population of younger, healthy individuals with induced shoulder pain following eccentric exercise. Although similar results have been observed in experimental and clinical trials [6], [7], [10], further validation of these findings to older and clinical populations is warranted. Third, our results are limited to those predictive factors included in our models. Other known predictors of resting pain such as depression were not included in these analyses. Furthermore, we did not include interaction terms within our model as we did not have specific hypotheses for interactions. Our models assessed only the main effects from each of the included predictors.

Additionally, we included only select measures of pressure and heat pain sensitivity and no other modalities. Currently, there is no consensus for pain sensitivity assessment, however multiple modality testing is encouraged and we included both static and dynamic pain sensitivity [13]. We did not include assessments of pain beyond 96-hours post-injury, but it is common for resolution of pain to occur for most individuals within this timeframe. Our pain outcome assessment was limited to pain intensity as compared to unpleasantness or other pain dimension measures. We did find some discrepancy in pain outcome between our study and the study by Dannecker and Sluka [5] where our activity-related pain measures did not elicit higher pain ratings than pain at rest. This may be due to the lack of specificity in our activity. In other words, we did not selectively stress the musculoskeletal tissue that was fatigued as was done by Dannecker and Sluka [5]. Further studies should assess whether induced shoulder pain can elicit higher pain ratings with selective movements as opposed to a general active or resisted motion.

### Conclusion

Our prediction models within an exercise-induced injury paradigm allowed us to assess whether pain sensitivity was related to pain outcome as a risk (pre-injury) factor or prognostic (post-injury) factor. Based on our findings, pain sensitivity and SHPR specifically is associated with activity-related pain, however further investigations are needed to validate these findings and their potential translation into clinical practice.

## Supporting Information

Data S1 (XLSX)
